# Metagenomic and metatranscriptomic analyses reveal minor-yet-crucial roles of gut microbiome in deep-sea hydrothermal vent snail

**DOI:** 10.1186/s42523-021-00150-z

**Published:** 2022-01-03

**Authors:** Yi Yang, Jin Sun, Chong Chen, Yadong Zhou, Cindy Lee Van Dover, Chunsheng Wang, Jian-Wen Qiu, Pei-Yuan Qian

**Affiliations:** 1 Department of Ocean Science and Hong Kong Branch of the Southern Marine Science and Engineering Guangdong Laboratory (Guangzhou), The Hong Kong University of Science and Technology, Hong Kong, China; 2 Southern Marine Science and Engineering Guangdong Laboratory (Guangzhou), Guangzhou, China; 3 Institute of Evolution and Marine Biodiversity, Ocean University of China, Qingdao, 266003 China; 4 X-STAR, Japan Agency for Marine-Earth Science and Technology (JAMSTEC), 2-15 Natsushima-cho, Yokosuka, Kanagawa 237-0061 Japan; 5 Laboratory of Marine Ecosystem and Biogeochemistry, Second Institute of Oceanography, State Oceanic Administration, Hangzhou, China; 6 Division of Marine Science and Conservation, Nicholas School of the Environment, Duke University, Beaufort, NC USA; 7 State Key Laboratory of Satellite Ocean Environment Dynamics, Second Institute of Oceanography, State Oceanic Administration, Hangzhou, China; 8 Department of Biology, Hong Kong Baptist University, Hong Kong, China

**Keywords:** Hydrothermal vent, Gut microbiomes, Metagenome, Metatranscriptome, Provannid snail

## Abstract

**Background:**

Marine animals often exhibit complex symbiotic relationship with gut microbes to attain better use of the available resources. Many animals endemic to deep-sea chemosynthetic ecosystems host chemoautotrophic bacteria endocellularly, and they are thought to rely entirely on these symbionts for energy and nutrition. Numerous investigations have been conducted on the interdependence between these animal hosts and their chemoautotrophic symbionts. The provannid snail *Alviniconcha marisindica* from the Indian Ocean hydrothermal vent fields hosts a *Campylobacterial* endosymbiont in its gill. Unlike many other chemosymbiotic animals, the gut of *A. marisindica* is reduced but remains functional; yet the contribution of gut microbiomes and their interactions with the host remain poorly characterised.

**Results:**

Metagenomic and metatranscriptomic analyses showed that the gut microbiome of *A. marisindica* plays key nutritional and metabolic roles. The composition and relative abundance of gut microbiota of *A. marisindica* were different from those of snails that do not depend on endosymbiosis. The relative abundance of microbial taxa was similar amongst three individuals of *A. marisindica* with significant inter-taxa correlations. These correlations suggest the potential for interactions between taxa that may influence community assembly and stability. Functional profiles of the gut microbiome revealed thousands of additional genes that assist in the use of vent-supplied inorganic compounds (autotrophic energy source), digest host-ingested organics (carbon source), and recycle the metabolic waste of the host. In addition, members of five taxonomic classes have the potential to form slime capsules to protect themselves from the host immune system, thereby contributing to homeostasis. Gut microbial ecology and its interplay with the host thus contribute to the nutritional and metabolic demands of *A. marisindica*.

**Conclusions:**

The findings advance the understanding of how deep-sea chemosymbiotic animals use available resources through contributions from gut microbiota. Gut microbiota may be critical in the survival of invertebrate hosts with autotrophic endosymbionts in extreme environments.

**Supplementary Information:**

The online version contains supplementary material available at 10.1186/s42523-021-00150-z.

## Background

The interaction between eukaryotic animals and microorganisms has resulted in numerous innovative symbiotic adaptations, especially in efficiency of energy utilisation. Such interplay in marine chemosynthetic ecosystems often supports distinctive microbial communities and may involve more than two players: the host animal could be symbiotic with microbes inhabiting different parts of the host body. For instance, gut bacteria contribute to digestive functions of the bone-eating snail *Rubyspira* [1]; alvinocaridid shrimps rely on epibionts associated with the gill chamber and the gut for both detoxification and nutritional intake [2]; provannid vent snails and bathymodioline mussels rely on gill endosymbionts for energy conversion and nutrition supply [3, 4]. A number of chemosymbiotic invertebrates from deep-sea ecosystems, including siboglinid tubeworms and several families of gastropods and bivalve molluscs, harbour microbes inside the host’s bacteriocytes while the host’s digestive systems is often reduced. These hosts rely mostly or entirely on the autotrophic chemosymbionts for energy and nutrition [5, 6]. Furthermore, laboratory evidence shows bathymodioline mussels are capable of filter-feeding and organic matter contributes to the diet of these mussels, suggesting some chemosymbiotic hosts have the potential to use multiple nutritional resources [7, 8]. Despite numerous investigations conducted on the interdependence between these animal hosts and their chemoautotrophic symbionts over the last decades [3, 4, 9, 10], intestinal functions and associated microbes received little attention [11]. Gut microbes are vital to host survival in mammals and insects, including through maintaining energy homeostasis, conferring metabolic capabilities, enhancing nutrient digestion and absorption and assisting with immunity development and activity [12]. They may also play important roles in chemosymbiotic animals.

*Alviniconcha* is a genus of chemosymbiotic vent snail in the family Provannidae with six species, five in the Pacific and one in the Indian Ocean [13]. These snails are endemic to hydrothermal vents and harbour chemoautotrophic endosymbionts in the gill epithelium [14]. The aggregated distribution of symbionts near the more exposed, outer surface of the bacteriocytes has been described as ‘semi-endosymbiotic’ condition [15, 16]. The Indian Ocean species *A. marisindica* hosts a single phylotype of *Campylobacterota* in its gills that is capable of oxidising sulphur, formate and hydrogen coupled with nitrate or oxygen reduction to produce cellular energy [8, 17]. In symbioses with a single major endosymbiont, the potential contribution of extracellular microbiota in the host gut has not been considered. Unlike many other invertebrate animals that rely on chemosynthetic endosymbionts and have lost most or all of the gut [5], such as siboglinid tubeworms and the awning-clam *Solemya reidi*, *Alviniconcha* retains a much reduced yet apparently functional gut and stomach [3, 18]. The stomach of *Alviniconcha* contains mainly mucus; lots of small white particles with walls and granular contents were found in some specimens, occasionally with mineral particles, sponge spicules, crustacean remains, and similar potential prey [18]. The intestine contains soft biogenic substances and mineral grains [3, 18]. These observations suggest that the stomach and intestine microbiota may contribute to the digestion of these foodstuffs and play nutritional, energetic and other physiological roles.

Integrated metagenomics and metatranscriptomics, which analyse the genomes and gene expression of the host and symbionts simultaneously, are powerful tools for deciphering symbiotic interactions [19, 20]. Here, an integrated metatranscriptomic and metagenomic approach was applied to investigate the gut microbial community and its interplay with the host snail by using *A. marisindica* from the Wocan hydrothermal vent field on the Carlsberg Ridge (CR), Northern Indian Ocean [21, 22].

## Results and discussion

### Gut microbial communities of *A. marisindica*

**Fig. 1:**
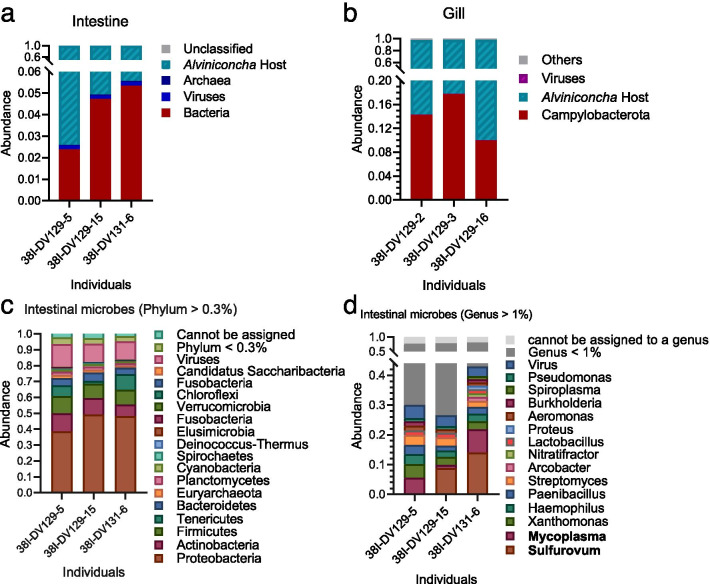
Abundance and community structure of microbiomes in the intestines of *Alviniconcha marisindica* from the Wocan vent field. Microbial taxonomic structures are deduced from the intestinal and gill metagenomes. The abundance of bacteria in **a** intestines and **b** gills are indicated in red colour. The microbial community compositions of intestines are displayed at the phylum and genus levels on the basis of Kaiju classification. **c** Phylum totalling > 0.3% and **d** genus totalling > 1% of the samples are shown

The metagenome assemblage of the intestinal content of *A. marisindica* using Illumina reads and subsequent taxonomic analyses of prokaryotic sequences was used to describe the intestinal microbial composition (Fig. 1; rarefaction curves for species abundance are shown in Additional file 1: Figure S1). In the intestinal metagenome of *A. marisindica*, the bacterial assembled contigs accounted for 2.6–5.6% of the total metagenomic sequences, much lower than that in the endosymbionts in the gill at 10.0–7.7% (Fig. 1a, b). Unlike the abundant single dominant campylobacterial endosymbiont in the gill (Fig. 1b), the intestinal tract harboured diverse microbial taxa (Fig. 1c, d) comprising 14 dominant genera (genera constituting > 1% of individual samples) from six phyla in three individuals of *A. marisindica* (Fig. 1d). The *A. marisindica* gut microbiome consisted of 8.74–49.30% Proteobacteria, 7.44–11.15% Actinobacteria, 8.98–10.79% Firmicutes, 1.89–9.75% Tenericutes, 3.20–4.42% viruses, and very few other microbes (Fig. 1c).

**Fig. 2:**
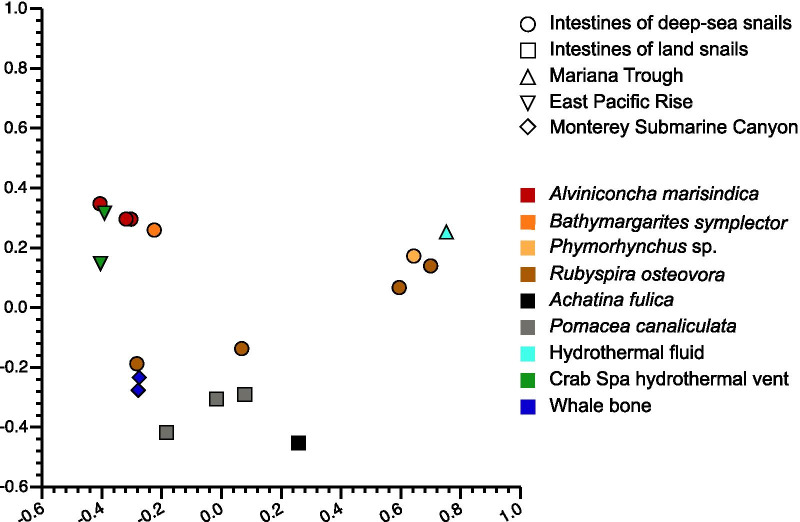
Principal coordinates analysis of three intestinal samples of *Alviniconcha marisindica*, six intestinal sample of other three deep-sea snails, four intestinal sample of two land snails and five environmental samples of three deep-sea habitats at the level of OTUs (97% sequence similarity). Different shapes represent the location of environmental samples and the snails from marine and land. Different colours represent the species names of all the snails and the types of deep-sea habitats. *A. marisindica* samples are clustered together with deep-sea scavenger *Bathymargarites symplector* and Crab Spa hydrothermal vent and well separated from other snails and environmental samples

Based on the available metagenomics and 16S rRNA amplicon data, intestines of *A. marisindica* exhibited a community structure different to the known gut microbiomes of other deep-sea snails from chemosynthesis-based ecosystems, including *Rubyspira osteovora*, *Bathymargarites symplector* and *Phymorhynchus* sp. [1], those of freshwater snail *Pomacea canaliculata* and land snail *Achatina fulica* [23, 24] and the environmental compositions of microorganisms in deep-sea whale fall, hydrothermal fluids and sediments [1, 25] (Additional file 2: Table S1). The four snails from deep-sea environments have different feeding modes: *Alviniconcha marisindica* depends largely on intracellular chemoautotrophic bacteria, *Rubyspira osteovora* feeds on whalebones, *Bathymargarites symplector* is a deposit feeder and *Phymorhynchus* sp. is a predatory and scavenging snail [1, 3, 26]. After the datasets of samples listed above were normalised on the basis of 16S rRNA sequences from Bacteria, Archaea and Fungi (RDP database), similarity analyses of microbial communities among these samples by PCoA (Bray–Curtis dissimilarity) revealed that environmental microbiome of the Crab Spa vent on the East Pacific Rise at 9°N [27, 28], the gut microbiomes of *A. marisindica* and *B. symplector* formed a distinct cluster and was separated from other samples (Fig. 2; ANOVA, F = 2.173, *P* = 0.00371). Environmental and snail gut microbiomes from deep-sea whale fall in Monterey Submarine Canyon were also separated from other samples. These results indicated that the principal environmental factors of different deep-sea habitats, such as vents versus whale falls, might have shaped different structure and function of microbial communities in both the environment and in the gut of animals living there. In particular, the *Mollicutes* and *Campylobacterota* in the intestine of *A. marisindica* made up a greater proportion of the total classified microbial sequences (average ~ 6.06% and 11.51%, respectively) than in the intestines of *A. fulica*, *Phymorhynchus* sp., *P. canaliculata* and hydrothermal fluid of Mariana Trough (~ 3.72%). In addition, *Mollicutes* and *Campylobacterota* were commonly identified in guts of the shrimp *Rimicaris exoculata* from the Mid-Atlantic Ridge hydrothermal vent sites [2], suggesting the broad distribution and importance of *Mollicutes* and *Campylobacterota* in guts of vent-endemic invertebrates.

At the genus level, *Sulfurovum* accounted for a large proportion of the gut microbes of *A. marisindica* (Fig. 1d), average ~ 7.87% of the total classified microbial sequences. The endosymbionts of gill epithelial cells in *A. marisindica* belong to *Sulfurovum* with one dominant ribotype [17], while two different ribotypes of *Sulfurovum* were found in the gut of *A. marisindica*. Based on the similarity of the 16S rRNA gene sequence, one gut ribotype is identical (> 99% similarity) with the gill endosymbiont, and the other gut ribotype shows 97.97% sequence similarity to the gill symbiont in the V6–V9 region of the 16S rRNA gene sequence. A previous study showed that campylobacterial epibionts in the gut and gill chamber of the vent shrimp *Rimicaris exoculata* were similar and suggested that the ones in the gut could be directly acquired from the environment or ingested from the gill chamber [2]. In *A. marisindica*, the intestines harboured multiple ribotypes of *Sulfurovum* compared to *Sulfurovum* ribotypes of the gills, further suggesting that the *Sulfurovum* in the gut were likely acquired from the environment, in addition to those from the gills. *Sulfurovum* is chemolithoautotrophic; it oxidises sulphur and thiosulfate (as electron donors) and uses the produced energy to fix carbon dioxide. A number of novel mesophilic, anaerobic chemosynthetic *Sulfurovum* have been isolated from hydrothermal sediments, vent plumes, and the tube of siboglinid polychaetes in vents [28–30]. In the gut microbiomes of deep-sea snails *R. osteovora*, *B. symplector* and *Phymorhynchus* sp., *Sulfurovum* is also one of the major components of their microbial communities [1]. Overall, *Sulfurovum* is abundant in the gut of *A. marisindica* and could be ingested from the gill and vent environment, the introduction of *Sulfurovum* may provide detoxification and nutritional intake for the host.

### Gut microbial co-occurrence pattern

**Fig. 3:**
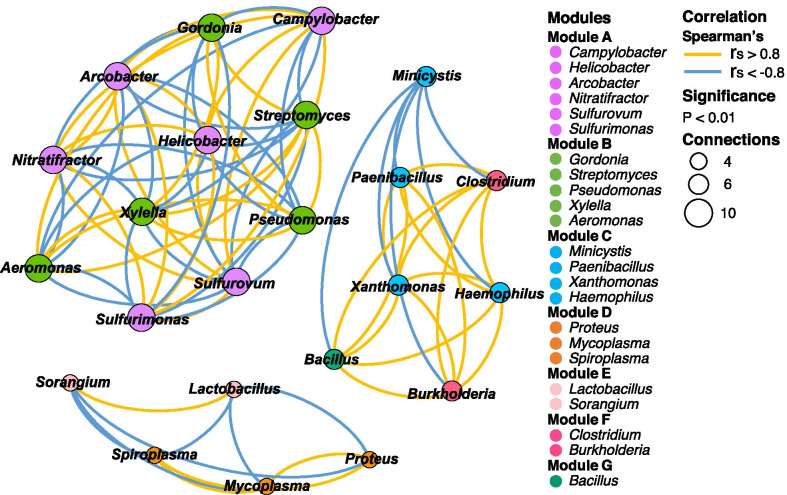
Correlation-based network of intestinal bacteria genera (relative abundance ≥ 0.5%) from three *Alviniconcha marisindica* individuals. Network analysis displays the intra-associations within each sub-community and inter-associations between sub-communities. Node size is proportional to the number of connections (i.e. degree of connectivity). Connection between nodes represents strong [Spearman correlation efficiency > 0.8 (yellow) or <  − 0.8 (blue)] and significant (*p*-value < 0.01) correlation. The same colour of nodes shows their highly modularised (clustered) property within the network

The gut microbiomes of three *A. marisindica* individuals exhibited significant non-random co-occurrence networks among component taxa (Fig. 3). The communities could be classified into seven modules differing mainly in the dominance of *Campylobacterota*, *Actinobacteria*, *Gammaproteobacteria*, *Bacilli* and *Mollicutes* (Fig. 3). In module A of *Campylobacterota*, six different genera were positively correlated. Amongst these genera, *Sulfurovum*, *Sulfurimonas* and *Nitratifractor* are chemolithoautotrophic bacteria commonly found in deep-sea hydrothermal environments. They function in sulphur- and/or hydrogen-oxidising respiration [14, 30–32], using elemental sulphur, thiosulfate and/or hydrogen as electron donors.

Module B, which is mainly composed of *Actinobacteria*, was the second largest group of gut microbes (Fig. 1c), and this module was negatively correlated with *Campylobacterota* in module A, likely due to their different physiological characteristics. In general, most *Actinobacteria* were aerobic and chemoheterotrophic [33], which may also apply to the *Actinobacteria* in the gut of *A. marisindica*. These *Actinobacteria* may compete for oxygen with *Campylobacterota*, which relies on oxygen for sulphur- and hydrogen-oxidising respiration.

In module C of *Gammaproteobacteria* and *Bacilli*, three genera of *Paenibacillus*, *Xanthomonas* and *Haemophilus* were positively correlated with one another and negatively correlated with *Minicystis*. Members of the genera *Xanthomonas*, *Paenibacillus* and *Haemophilus* are known as plant-, human- or insect-associated microorganisms and many of them are pathogenic. *Xanthomonas* and *Paenibacillus* species in animals’ guts have the ability to degrade non-digestible carbohydrates such as the cell-wall polysaccharide of plant-like protists and cellulose using xylanases and cellulases [34, 35]. *Minicystis* species has been identified as omega-3 fatty acid (FA) producer [36]. Omega-3 FA is a predominant polyunsaturated FA, with high concentrations found in marine snails [37]. They are also essential for physiological processes. In addition, putative omega-3 FA-producing bacteria in the gastrointestinal tracts of marine fishes contribute omega-3 FAs to the host in general [38] and have integral roles in regulating membrane fluidity in response to temperature fluctuations [38–40]; this may also apply to vent-restricted *A. marisindica* living in relatively instable environmental conditions.

Module D was mainly composed of *Proteus* and *Mollicutes*-related bacteria that are commonly considered as opportunistic pathogens in a wide variety of animals. This module showed a significant negatively correlation with module E, which included *Lactobacillus* that are typically intestinal probiotics [41] (Fig. 3). In addition, lactic acid bacteria (LAB), which are considered vital for maintaining the gut ecological balance [42], accounted for at least ~ 2.70% of the *A. marisindica* gut microbiome. Mixed LAB exhibited a mutualistic relationship with the host; they have the potential to prevent pathogens from causing intestinal infections and are vital for food digestion and energy provision to the host [41–43]. Various LAB were found to co-exist in the intestine of *A. marisindica*, including *Lactobacillaceae* (1.29–1.55%), *Streptococcaceae* (0.20–0.43%) and *Enterococcaceae* (0.25–0.34%). This finding further suggested a potential ecological balance in the *A. marisindica* gut microbiome.

Although microbial sequences obtained from the intestinal metagenome data of *A. marisindica* were in low abundance (2.61–5.57%), modules of co-occurring microorganisms could be identified from the 3 individuals. Positive and negative associations suggest that their interactions may shape the microenvironment in the intestine of *A. marisindica*; coexisting microbial species in the gut may have similar, complementary, or competitive ecological functions.

### Essential functions of gut microbiome

**Fig. 4:**
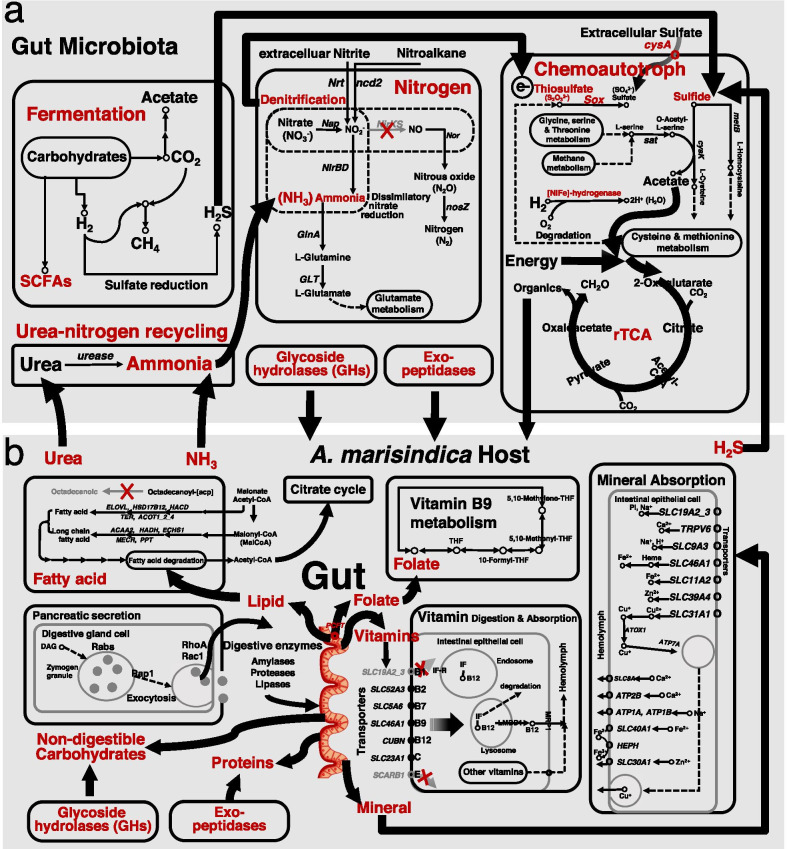
Overview of meta-pathways of *Alviniconcha marisindica* and its gut microbiome. Metabolic pathways of different organisms, including **a** the gut microbiome and **b** *A. marisindica* host, are presented to reveal the functional contribution of gut microbiomes to the host. Important metabolic intermediates and complementary intermediate products are indicated in red colour

The gut microbiome of *A. marisindica* contained ~ 3881–5389 annotated genes exhibiting a wide metabolic spectrum. In this study, the microbial genes were analysed to reveal microbial pathways associated with the metabolism of host-ingested substances, including carbohydrates, proteins, vitamins and minerals. These functions were distinct from, and likely complement the activities of host enzymes in the digestive gland (DG) and intestine, including essential functions of organic matter digestion and nutrient absorption. Accordingly, genes involved in substrates transportation, energy conversion, macromolecular digestion, and absorption were enriched in the intestine (Additional file 1: Figure S2), especially for genes involved in lipid metabolism, indicating the metabolic capacity and activity of intestines. In addition, the gut microbiome of *A. marisindica* was capable of ammonium assimilation, indicating a role of the guts in recycling the host’s metabolic waste (Fig. 4a) and in producing other metabolites essential for intestinal homeostasis, such as bacteriocins, short-chain FAs (SCFAs), and quorum-sensing autoinducers [44].

Glycoside hydrolases of saccharolytic bacteria in the gut microbiome of *A. marisindica* (Additional file 2: Table S2) can break down sugars, including host-indigestible carbohydrates, resulting in the production of important metabolites such as vitamins and SCFAs. Most SCFAs are the end products of gut microbial anaerobic metabolism via digestion of the dietary carbohydrates, such as acetate, propionate, and butyrate for nutrition in aquatic animals [45, 46]. In addition, microbial fermentation produces SCFAs together with the gases CO_2_ and H_2_, which could be used by chemolithoautotrophic microbiota in the gut of *A. marisindica*. For example, *Sulfurovum* and *Sulfurimonas* could generate energy through hydrogenotrophic energy metabolism and fix CO_2_ into organics. Evidence showed that meta-pathways of intestinal *Campylobacterota* contain genes encoding [NiFe]-hydrogenases for hydrogenotrophic metabolism, the Sox system for thiotrophic metabolism, and the reverse tricarboxylic acid cycle for carbon fixation (Fig. 4a). Therefore, these gut microbes of *A. marisindica* have the potential of chemoautotrophy, consistent with the metagenomic findings described above. In addition, the genera *Desulfitobacterium* and *Desulfovibrio* in the gut of *A. marisindica* are sulphate-reducing microorganisms that could produce H_2_S which can then be used by thiotrophic gut bacteria, such as *Sulfurovum* and *Sulfurimonas*.

Nitrogen is essential for bacterial survival. Genes encoding for dissimilatory nitrate reduction and ammonium assimilation are found in the metagenome of gut microbes (Fig. 4a). Genes encoding for ureases are also found in gut microbiomes, urea produced by the host could be hydrolysed to ammonia by these urease-producing bacteria and the ammonia in turn could be used for protein metabolism (Fig. 4a). The occurrence of urea-nitrogen recycling suggests that the gut microbiomes play an important role in the nitrogen balance of *A. marisindica*. In addition, genes encoded the sialate O-acetylesterase (SIAE) were highly expressed in the meta-transcriptome of intestinal microbiomes, indicating that active sialic acid (Sia) degradation in the intestine of *A. marisindica*. Sialic acids are prominent outermost carbohydrates of the intestine; they are important components of the mucus layer [47]. A pathway for the transport and catabolism of Sia is encoded in the metagenome of intestinal microbiomes, indicating Sia could be utilised as a carbon source for heterotrophic microbes in the gut. The intestinal microbiome contains biosynthetic pathways for four vitamins and 10 amino acids, including six amino acids and three vitamins that cannot be synthesised by the host (Table 1), indicating that gut microbiomes could provide essential nutrients for the host.

**Table 1:** Nutrient biosynthesis capability of gut microbiomes of *Alviniconcha marisindica* from Wocan site, showing the nutrients with and without complete biosynthesis pathways in the metagenomes of gut microbiomes

Nutrients	Description	Gut microbiota	*A. marisindica*
**Biosynthesis of amino acids**			
NEFAAs	bA, E, Q, R	+	+
NEFAAs	A, C, D, G, M, N, Orn, P, S, T	−	+
EFAAs	F, H, I, K, L, Q, R, V	+	−
**Biosynthesis of vitamins and cofactors**			
Vitamin B1	Thiamine	+	−
Vitamin B2	Riboflavin	+	−
Vitamin B3	Nicotinate and nicotinamide	+	+
Vitamin B5	Pantothenate	+	−
Vitamin B6	Pyridoxine	−	−
Vitamin B7	Biotin	−	−
Vitamin B9	Folate	−	−
Vitamin B12	Cobalamin	−	−
Vitamin K1	Phylloquinone	−	−
Vitamin K2	Menaquinone	−	−
Coenzyme A	CoA	−	+
Coenzyme Q	Ubiquinone	−	−
	Protoheme (heme)	−	+
	Siroheme	−	−

Amino acids (black colour): A—Alanine, bA—β-Alanine, C—Cysteine, D—Aspartate (aspartic acid), E—Glutamic acid, F—Phenylalanine, G—Glycine, H—Histidine, hypoTa—Hypotaurine, I—Isoleucine, K—Lysine, L—Leucine, M—Methionine, N—Asparagine, Orn – Ornithine, P—Proline, Q—Glutamine, R—Arginine, S—Serine, T—Threonine, Ta—Taurine, V—Valine, W—Tryptophan, Y—Tyrosine; Vitamins/cofactors (red colour): B1—Thiamin, B2—Riboflavin, B3—Nicotinate and nicotinamide, B5—Pantothenate, B6—Pyridoxine, B7—Biotin, B9—Folate, B12—Cobalamin, K1—Phylloquinone, K2—Menaquinone, CoA—Coenzyme A, CoQ—Coenzyme Q (ubiquinone); Complete and missing pathways are indicated by a ‘ + ’ and ‘−’, respectively

Co-abundance and meta-pathway analyses indicated that the gut microbiome likely plays critical ecological roles via bacterial interdependencies and mutual cooperation to maintain microbial homeostasis and provide the host with symbiont metabolites that serve as host nutrients. In addition, the gut microbiomes of *A. marisindica* were found to produce large exo-enzymes for hydrolysing various macromolecules. Thus, they have the potential to digest and supply exogenous nutrients to the host. This finding warranted further investigation on the mutual cooperation of *A. marisindica* holobiont (that is, involving the *A. marisindica* host and its gill endosymbiont) in the production of nutrients.

### Host–gut microbiome cooperation

In the study of *A. marisindica* holobiont [17], the gill endosymbionts provide plenty of nutrients to *A. marisindica* although they still cannot synthesise some nutrients, i.e. two amino acids of taurine and hypotaurine and seven vitamins/cofactors of thiamin, pyridoxine, folate, cobalamin, phylloquinone, menaquinone, and ubiquinone. Neither *A. marisindica* nor its endosymbionts can synthesise folate, but the host transcriptome contained a complete folate metabolism pathway (including one carbon pool by folate, Fig. 4b), indicating that the host has the ability to use folate and it may be essential to supporting *A. marisindica*. Furthermore, *A. marisindica* holobionts cannot synthesise octadecanoic acid and other 10 typical unsaturated FAs [17] but the host harboured a complete pathway of FA metabolism (Fig. 4b). Therefore, the nutrients that cannot be produced by the *A. marisindica* holobiont are likely supplemented by taking food from the environment [3, 18], followed by intestinal digestion and absorption of substances [48]. This is analogous to the situation in bathymodioline mussels, which also rely on the mixotrophic nutritional strategy of suspension-feeding and endosymbiosis [7, 8], and is in contrast to our understanding of nutrition in other chemosymbiotic animals that entirely rely on endosymbiont nutritional supply [4, 6, 20, 49].

**Fig. 5:**
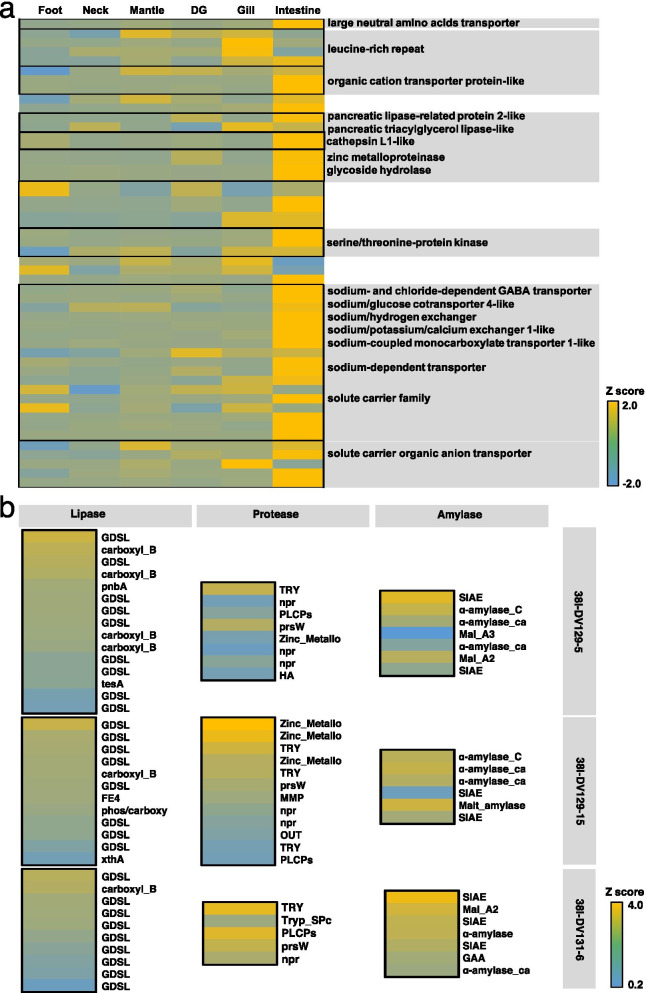
Transcriptional activity of genes participating in the nutrient digestion and absorption of Wocan *Alviniconcha marisindica*. Heat map of transcriptional activity of genes that **a** encode specialised transport proteins and various hydrolases, including proteases, glycoside hydrolase, and peptidoglycan recognition proteins (PGRPs) in the foot, neck, mantle, digestive gland (DG), intestine and gill tissues and are involved in **b** gut microbial exo-hydrolase biosynthesis for intestinal nutrient digestion in *A. marisindica*

In the present study, the *Alviniconcha* transcriptome encoded metabolic pathways within lysosomes, endosomes and phagosomes which all served as the main digestive compartment of host cells. Accordingly, functional enzyme-encoding gene distribution in the *A. marisindica* genome showed that the host contains numerous genes responsible for key hydrolases [17] and the genes for intestinal digestion and absorption of ingested dietary components, such as carbohydrate, fat, protein and vitamin are active (Fig. 4b). For example, genes encoding various large proteases, bile salt-activated lipase, and glycosyl hydrolases were highly expressed in the intestine and genes encoding a gastric intrinsic factor and a bile acid transporter were highly expressed in the digestive gland (Fig. 5a), supporting the active digestion of macromolecular nutrients in the intestine. In addition, genes encoding specialised transport proteins, such as proton-coupled folate transporter (*SLC46A1*), taurine transporter (*SLC6A6*), cationic amino acid transporter (*SLC7A6*), and ferritin (*FTH1*), were expressed actively in the intestine (Fig. 4b and Fig. 5a). This expression may help the intestinal absorption of folate, amino acids, and minerals that could not be generated by the *A. marisindica* holobiont. Large numbers of highly expressed genes in the intestine showed that the reduced gut still has functional specialisations responsible for effective and regulated nutrients transport. The number of differentially expressed genes (DEGs) in the intestine, with their biological coefficient of variation (BCV), is shown in the Additional file 1: Figure S3. The annotation of highly expressed genes in the intestine of *A. marisindica* were shown in Additional file 3: Dataset S1. On the basis of the DEGs, functional enrichments of GO terms/KEGG pathways were also identified in the intestine of *A. marisindica*. In the intestine, genes involved in vacuolation, hydrolases activity, organic hydroxy compound metabolic processes, and transmembrane transport were enriched (Additional file 1: Figure S2), indicating its activity in nutrients digestion and absorption.

Intestinal microbial enzymes contain 30.6%–34.7% hydrolases, 26.7%–28.2% transferases and 9.6%–17.4% oxidoreductases. The three main types of hydrolases secreted by gut microbiomes are glycoside hydrolases (GHs), which could break down complex carbohydrates (e.g. cellulose and chitin); protease (including aminopeptidases) and esterase (mainly lipases). Genes involved in intestinal microbial hydrolases were extracted from the metagenome and their annotations and expression levels are summarised in Additional file 2: Table S2. In *A. marisindica*, the digestive exoenzymes produced by gut microbes may help with the enzymatic digestion of intestinal substances. For example, genes encoding α-amylase, which breaks down long-chain saccharides, were abundant and highly expressed in the gut microbiomes (Fig. 5b) that may help degrade the glycogen produced by animal remains found in the gut [18]. Trypsin and zinc-dependent metalloprotease are major exoproteases (Fig. 5b) necessary for protein absorption. GDSL esterases/lipases (Fig. 5b) are the major lipolytic enzymes for carbon source provision. Proteins and lipids are the main components of animal remains and mucus found in the stomach and gut of *Alviniconcha* snails [18]. In particular, genes encoding GHs were found abundantly in the gut of *A. marisindica* (Additional file 2: Table S2) and they have important roles in aiding the digestion of dietary carbohydrates. The above results indicated abundant digestive exoenzyme production in the gut of *A. marisindica*. Complementary to the host metabolic capacities, the intestinal microbiota provides additional enzymes that are not encoded by *A. marisindica*. For example, comparative analyses among the available lophotrochozoan genomes revealed *A. marisindica* lacked the enzymes (e.g. cellulases) to degrade the bulk of dietary fibres [17]. The gut microbial metagenome and metatranscriptome data showed that genes encoding cellulases, xylanase/chitin deacetylase, glucanases, phosphorylase and some other GHs were found extensively in Proteobacteria (*Dickeya*, *Xanthomonadales* and *Cellvibrio*), Actinobacteria, Firmicutes, Tenericutes, Chloroflexi and Bacteroidetes (Cytophagia) to form multi‐enzyme complexes that help with the host digestion of ingested carbohydrates such as remains of crustaceans and similar things [18]. Moreover, oligoendopeptidase F (*pepF1*) and alginate lyase (*algL*) are not encoded by the host [17] but found encoded by the gut microbiomes and they become complementary for food digestibility in *Alviniconcha* snail. The results indicated the ability of intestinal microbiomes to assist the host’s intestinal digestion of food for nutrient absorption. All annotated information of gut microbiomes is shown in Additional file 4: Dataset S2.

Host–gut microbiome cooperation in the production and absorption of nutrients is newly revealed here for the vent snail *A. marisindica*, indicating that the maintenance of the *A. marisindica* symbiosis is also likely mediated by feeding and the gut microbiome, and not only depends on gill endosymbionts.

### Maintenance of gut microbiota

**Fig. 6:**
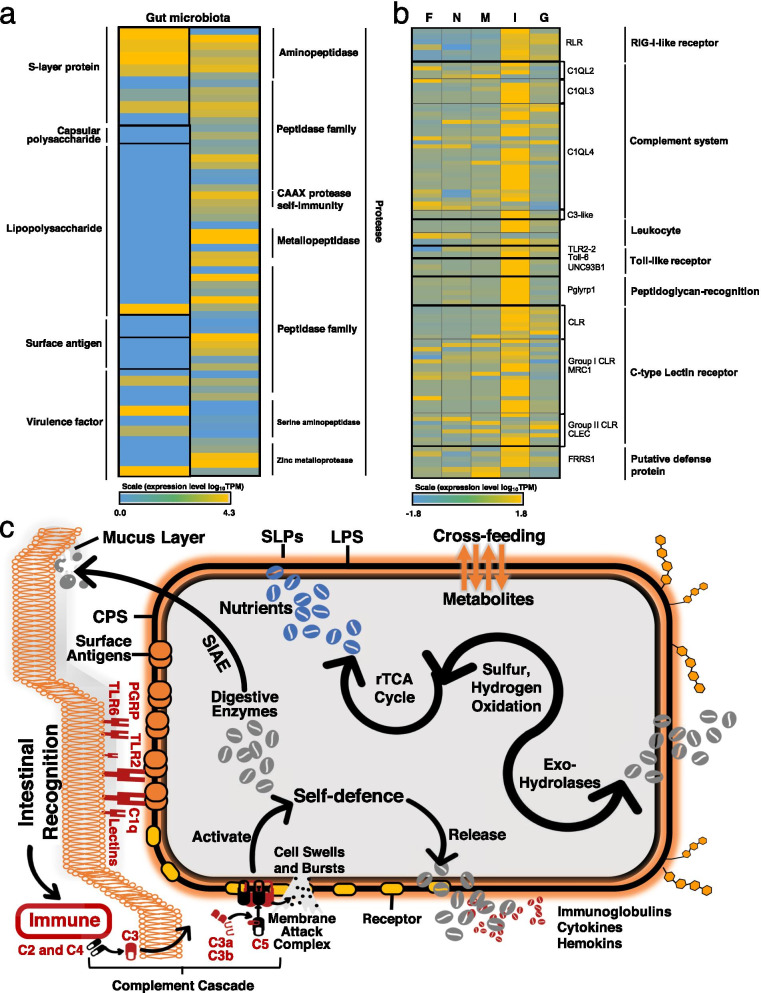
Maintenance of microbiomes in the gut of *Alviniconcha marisindica*. Heat map of the transcriptional activity of genes that **a** participate in bacterial surface-associated virulence factors, surface modification, protease synthesis and secretion in the gut microbiome and are **b** involved in host innate immunity in the foot (F), neck (N), mantle (M), intestine (I) and gill (G) tissues, showing an immune-expression profile of the gut regulated by its inhibited microbiomes. Each grid in the heat map represents an identified gene. The colour represents the gene expression (based on normalised TPM values of the selected tissues). The annotated gene names and their functional classifications are listed on the two sides. **c** Host–microbiota homeostasis is maintained by the host’s immune compartmentalisation and bacterial counter-defence. All pattern recognition receptors (PRRs) and pathogen-associated molecular patterns (PAMPs) shown here are identified from the transcriptome data. SLPs, surface layer proteins; LPS, lipopolysaccharide; CPS, capsular polysaccharides; SIAE, sialate O-acetylesterase; PGRPs, peptidoglycan recognition proteins; TLR, toll-like receptor; C1q, complement component 1q

Genes involved in the assembly of bacterial capsular polysaccharides (CPs), surface layer (S-layer) proteins (SLPs), bacterial lipopolysaccharide (LPS) and other surface-associated antigens were found in gut microbiomes such as *Campylobacterota*, *Clostrida*, *Mollicutes*, *Bacilli*, *Erysipelotrichia*; they are responsible for the bacterial adhesion to the intestinal epithelium and they activate the complement system [50, 51]. LPS is often the first target of host immune system and it could induce a strong host immune response [50, 52]. Although CPs and SLPs could be recognised by the host as immunodominant antigens [51, 52], they are bacterial physical “cloaks” that protect intracellular bacteria from many of host’s defences [53, 54]. Surface-layer glycoprotein variation in the gut microbiome was evident from the differential expression of S-layer genes, a type of antigenic variation responding to the lytic activity of the host immune system (Fig. 6a). In the gut metagenome of *A. marisindica*, genes encoding for SIAE were found in Cytophagia (e.g. *Leeuwenhoekiella*) and Sphingobacteriia in the phylum Bacteroidetes; their high expression levels (Fig. 5b) indicate active sialic acid degradation in the intestine of *A. marisindica*. Sias are prominent carbohydrates of the intestinal mucus layer [47]. The mucus layer is the interface between the gut microbiome and the host. Thus, Sia breakdown is a mechanism for intestinal bacterial encroachment and survival. Gut-specific bacteria could bind to and degrade mucin glycans as a nutritional source and produce microbial metabolites that facilitate growth of other microbial species [47].

The intestine harboured much lower abundance of bacteria than the gill (Fig. 1a, b), but the intestinal immune response was stronger than that of the gill (Fig. 6b). The microbes in animals have the ability to control immune gene transcriptional activity [20, 55]. Host immune responses show the interaction of microbiota with the host. Pattern recognition receptors (PRRs) are crucial to the innate immune system and they could be divided into membrane-bound PRRs and cytoplasmic PRRs. Genes encoding membrane-bound C-type lectin receptors (CLRs) and cytoplasmic RIG-I-like receptors (RLRs) were active in the intestine (Fig. 6b). Toll-like receptors (TLRs) recognise structurally conserved molecules derived from microbes and activate immune responses. Genes encoding membrane-bound TLR2 and TLR6 were active in the intestine (Fig. 6b), indicating a distinct TLR-expression profiles in the intestine that recognise inhabited microbes. Once the host recognised the microbes in the gut, the component cascade is activated, as indicated by the highly expressed complement component 1 complex (*C1*) and complement C3 (*C3*, Fig. 6b), which both attack the microbe’s cell membrane and eliminate microbes to control bacterial infections [56] (Fig. 6c). Differentially expressed genes of the gut immune system compartmentalise the intestinal bacteria; these genes are essential for mutual coexistence of *A. marisindica* and its gut microbiome. The host’s immune compartmentalisation and bacterial counter-defence cooperate to maintain the host–gut microbiome mutualism (Fig. 6c).

Functional analyses of gut microbiomes indicated that the microbiota have a considerable effect on intestinal nutrient digestion and absorption and host immune system stimulation that help defend against pathogens. The digestion processes in the intestine following the action of bile acid and pancreatic and intestinal enzymes are essential for nutritional homeostasis in *Alviniconcha* holobiont. Thus, even if the gut microbiome has a lower cell abundance than the endosymbionts in the gill, it is important—and likely critical—to the host fitness and survival. Gut microbial ecology and the interplay of this ecology with the host metabolome contribute to meet the nutritional and metabolic demands of *Alviniconcha* holobiont and they help shape the distinctive microenvironments and physiology of the intestine. These results complement the recent study of the symbiosis between this snail and its gill endosymbiont [17] and enhance our understanding of the contribution of gut microbes to the success of chemosymbiotic snails.

## Conclusions

In this study, the microbial community structure and their relative abundance in the gut of the deep-sea vent snail *Alviniconcha marisindica* were illustrated. More importantly, the metabolite-enabled mutualistic interaction between the host and its gut microbiota was described through gene functional and expression analyses. The energy requirements of *A. marisindica* were potentially sustained by feeding and through its functionally versatile gut microbiome. The findings advanced general understanding of the mechanisms of animals surviving in extreme chemosynthetic ecosystems. Furthermore, the gut microbiome may have evolved in parallel with host immune systems and has strategies for coping with the host’s immune responses in the gut. Potential interactions of the gut and its microbiome in deep-sea vent invertebrates that primarily rely on endosymbiosis are reported for the first time. These interactions are likely important for the efficient and successful life of these animals at deep-sea vents.

## Methods

### Sample collection and nucleic acid preparation

*Alviniconcha marisindica* were collected from the Wocan vent field on the CR of the Northwestern Indian Ocean at a water depth of 2,919 m (60.53°E, 6.36°N). Sampling was conducted using the human-occupied vehicle (HOV) *Jiaolong* onboard R/V *Xiangyanghong 9* on March 19, 2017. Snails were placed into an insulated ‘BioBox’ with a closed lid by using a manipulator to minimise changes in water temperature. Around 2.5 h was needed for HOV *Jiaolong* to be recovered on deck. Once the snails were on-board the research vessel, all specimens were immediately flash-frozen in liquid nitrogen and then transferred to − 80 °C freezer for storage. The external morphology of a complete *Alviniconcha* individual preserved in absolute ethanol is shown in Additional file 1: Figure S4. The internal morphology was observed under a Leica MZ9.5 stereozoom microscope and the snails were dissected to isolate their radula. Radular morphologies of two specimens were imaged using scanning electron microscopy (SEM, Additional file 1: Figure S5). Radular sacs were dissected from the body cavities, stored in pure ethanol and then treated with half-strength commercial bleach, leaving the clean radular teeth. Subsequently, the radula for SEM was rinsed in MilliQ water and dehydrated by increasing the concentration of ethanol solution (20%, 40%, 60%, 75% and 100%). The dehydrated radula was air-dried and then SEM observation was undertaken, uncoated at 15 kV using a Hitachi TM-3000 SEM.

Three frozen snails were thawed in RNAlater (Invitrogen, USA) on ice and the intestine was carefully dissected for total DNA and RNA extraction. The total DNA of the intestine was extracted using the E.Z.N.A. Mollusc DNA Kit (Omega Bio-tek, Georgia, USA) and then purified using Genomic DNA Clean & Concentrator-10 Kit (Zymo Research, CA, USA) in accordance with the manufacturer’s protocol. The total RNA was extracted using TRIzol (Invitrogen, USA) following the manufacturer’s protocol and prepared for RNA-Seq. Nucleic acid quality was evaluated using agarose gel electrophoresis and a BioDrop µLITE (BioDrop, Holliston, MA, USA). Nucleic acid concentrations were quantified using a Qubit fluorometer v3.0 (Thermo Fisher Scientific, Singapore).

### Library construction and sequencing

The total DNA of the intestine was submitted to Illumina sequencing platform. A library with a 350 bp insert size was constructed following the standard protocol provided by Illumina (San Diego, CA, USA). After paired-end sequencing of the library was conducted at Novogene (Beijing, China), approximately 12 Gb of Illumina Novaseq reads with a read length of 150 bp were generated from each of the three intestine specimens for metagenome analysis.

As the RNA of the intestine includes sequences from the host and microbes, 250–300 bp insert stranded-specific library of each intestine specimen was constructed using Ribo-Zero Magnetic Kit to sequence eukaryotic and microbial RNAs. The meta-transcriptome sequencing of the intestine was conducted on the Illumina Novaseq platform at Novogene to produce 150 bp paired-end reads. Approximately 10 Gb of reads were generated from each intestine specimen.

### Microbial metagenome assembly, annotation, and functional analyses

For microbial metagenome assembly of the intestine, Trimmomatic v0.39 [57] and FastUniq [58] were used to trim the Illumina reads and remove duplicates introduced by polymerase chain reaction (PCR) amplification. Four metagenomes were assembled from the eukaryotic reads obtained from each snail individual and the merged dataset of all three individuals. The scaffolds of these metagenomes were individually aligned to the host’s whole genome [17] by using minimap2 [59], which returned the alignment rates of 95–97% and thus confirmed that they originated from *Alviniconcha marisindica* host. The host’s interference in the analysis of intestinal content was minimised by removing reads that aligned to the host genome by using Bowtie2 [60] before the assembly. The abundance and taxonomic classification of intestinal metagenomic microbial sequences were carried out using Kaiju [61] on the basis of completely assembled and annotated reference genomes of Archaea, Bacteria and Viruses from the NCBI RefSeq database. The clean reads left were assembled using MEGAHIT [62] and metaSPAdes v3.13.1 [63] respectively. Prodigal v2.6.3 [64] was used to predict and translate the coding sequences in the intestinal metagenome. Then, BLASTp was used to align the candidate sequences to the NCBI NR protein database and the taxonomic assignment of each protein was imported to MEGAN v5.7.0 [65] by using the lowest common ancestor (LCA) method with the parameters of Min Score 50, Max Expected 0.01, Top Percent 5 and LCA Percent 100. On the basis of the taxonomic results, the microbial protein sequences were selected for further gene functional analysis, following the gene annotation pipeline described above. Blast2GO [66] and EggNOG mapper [67] were applied to assign GO and COG terms to the intestinal prokaryotic protein sequences, KAAS [68] was used to annotate KEGG numbers of prokaryotic protein sequences by using the single-directional best hit method. Specific metabolic pathways of multiple microorganisms were combined to reconstruct microbial meta-pathways through the KEGG mapper. The associated species annotation of the corresponding protein-encoding genes provides a reference for determining which bacteria contribute to a given enzymatic reaction in the meta-pathway.

### Statistical analyses

Data were statistically assessed by using the results from the metagenomics RAST server (MG-RAST) to compare the microbial communities in the gut of *Alviniconcha* snails with other snails or environments [69]. Whole genome sequencing (WGS) and 16S amplicon samples were mapped against 16S rRNA database RDP, the number of hits of the reads of 16S amplicon and WGS samples mapped against the RDP database were calculated respectively. After automatic normalisation by MG-RAST, the relative abundance of microbial taxonomic groups detected in the WGS and 16S amplicon samples can be compared. The α-diversity of the annotated intestinal metagenomic samples could be estimated from the distribution of the species-level annotations. Annotated species richness is the number of distinct species annotations in the combined MG-RAST data set. Principal coordinates analysis (PCoA) of the microbial communities and Rarefaction curve of annotated species richness were calculated in MG-RAST. Comparison analysis was performed using paired metagenomic samples and gut metagenomics of other snails and microbiomes of deep-sea vent environments from the MG-RAST server were collected for comparison (Additional file 2: Table S1). Differences between the samples were assessed using ANOVA.

### Microbial co-occurrence analysis

Network analysis was conducted on the basis of relative abundance of microorganisms in three intestinal samples. The abundance and taxonomic classification of intestinal metagenomic microbial sequences were carried out using Kaiju [61]. To reduce the complexity of the datasets, only relative abundances higher than 0.5% were included. All pairwise Spearman’s rank correlations between genera of three intestinal samples were calculated in the R package ‘picante’. Only robust (r > 0.8 or r <  − 0.8) and statistically significant correlations (*P* < 0.01) were shown in the network. Network visualisation and modular analysis were conducted using Gephi v0.9.2. Nodes in the reconstructed network represent the genera in three intestinal samples, whereas the edges (connections) correspond to a strong (r > 0.8 or r <  − 0.8) and significant (P < 0.01) correlation between nodes. The clustering coefficient was 1.0 (the degree to which nodes tend to cluster together) and the modularity index was 0.5 (values > 0.4 suggest that the network has a modular structure [70]).

### Gene expression level quantification

For the intestinal meta-transcriptome data, the raw reads were trimmed with Trimmomatic v0.39 [57]. A Salmon index built for the transcripts of the host was obtained and translated from its complete genome data [17] and then the trimmed reads were quantified directly against this index and expressed in transcripts per million (TPM) by Salmon [71]. The read counts for genes were also included in the quantification results. For gut microbiome, the same pipeline was used, with a Salmon index built for the transcripts of microbes obtained and translated from their metagenome data; then, the trimmed reads were quantified directly against this index and expressed in TPM by using Salmon [71]. The gene expression levels of the intestine and its microbiome were produced by using this quantification method and the consistency of gene expression levels for the intestine from three snail individuals indicated the high accuracy of the transcript-level quantification method.

Transcriptome data from the foot, neck, mantle, DG and gills of *A. marisindica* [17] were compared to show gene differential expression in the intestine. DEGs were determined by DESeq2 using the normalisation method of Loess, a minimum read count of 10 and a paired test (n = 5). Genes considered to be highly expressed in the intestine were overexpressed with over two-fold changes and FDR < 0.05 when compared with other tissue types. WEGO (http://wego.genomics.org.cn/cgi-bin/wego/index.pl) was used to plot GO annotations of highly expressed genes in the intestine. Statistically overrepresented GO terms in the intestine were identified through the topGO package in R session (10.18129/B9.bioc.topGO). The GO enrichment network was visualised using the Cytoscape application [72]. The DEGs of the intestines are shown Additional file 3: Dataset S1.

## Supplementary Information

**Additional file 1**. **Figure S1.** Rarefaction curves for gut microbial samples of three *Alviniconcha marisindica* individuals from the Wocan vent field. **Figure S2**. Gene Ontology (GO) enrichment network of differentially expressed genes (DEGs). The significantly (p-value < 0.01) enriched GO terms of selected highly expressed genes in the intestine of *Alviniconcha marisindica* are clustered in accordance with their functional category. The connecting pairs of nodes showing the intra-cluster and inter-cluster similarities of enriched terms. The colour code represents different cluster annotations. Each node represents an enriched term. **Figure S3.** Differentially expressed genes (DEGs) in the intestine and gill of *Alviniconcha marisindica*. Volcano plot and biological coefficient of variation (BCV) plot of DEGs in the intestine are identified by DESeq2 analysis. The log10 (FDR corrected p-values) are plotted against the log2 (FC) in gene expression. Upregulated genes by twofold or more and with a FDR corrected p-value < 0.05 are marked as blue dots, whilst down-regulated genes (FRD ≤ 2 with P < 0.05) are marked in red colour. **Figure S4.** Photograph of snail *Alviniconcha marisindica* collected from the Wocan hydrothermal field (WHF) and stored in absolute ethanol. **Figure S5**. SEM images of radula. Overview: (**a**) *Alviniconcha marisindica* (individual 01); scale bar = 300 μm (**b**) *A. marisindica* (individual 01); scale bars = 200 μm. Central and lateral teeth close-up: (**c**) *A. marisindica* (individual 01); scale bars = 200 μm (**d**) *A. marisindica* (individual 02); scale bars = 200 μm. Marginal teeth close-up: (**e**) *A. marisindica* (individual 02); scale bars = 30 μm.

**Additional file 2**. **Table S1.** Metagenomic and 16S rRNA amplicon data of intestines of *Alviniconcha marisindica* from the Wocan field, intestines of giant land snails, intestines of deep-sea bone-eating, scavenger and predatory snails and environmental samples from deep-sea habitats. **Table S2.** Annotation and expression levels of genes involved in encoding representative exo-hydrolases in intestinal microbiomes of three *A. marisindica* individuals from the Wocan site.

**Additional file 3**. **Dataset S1** The annotation of highly expressed genes in the intestine of *A. marisindica*.

**Additional file 4**. **Dataset S2** The annotation of genes predicted from the metagenome of intestinal flora from three snail individuals.

## Data Availability

All raw sequencing data generated in the present study are available from NCBI via the accession numbers SRR11781645, SRR11781642, SRR11781640, SRR11781646, SRR11781639, SRR11781643 and BioSamples via accession numbers SAMN14907815, SAMN14907816, SAMN14907817. The datasets supporting the Conclusions of this article are included within the article (and its Additional files 1–4).

## Abbreviations

BCV: Biological coefficient of variation
LPS: Lipopolysaccharide
CLRs: C-type lectin receptors
CR: Carlsberg Ridge
CPs: Capsular polysaccharides
C1q: Complement component 1q
DG: Digestive gland
DEGs: Differentially expressed genes
FA: Fatty acid
GHs: Glycoside hydrolases
LAB: Lactic acid bacteria
PGRPs: Peptidoglycan recognition proteins
rTCA: Reverse tricarboxylic acid
RLRs: RIG-I-like receptors
Sia: Sialic acids
SLPs: Surface layer proteins
SIAE: Sialate O-acetylesterase
TLR: Toll-like receptor
