# Supplementary material for: Metagenomic and metatranscriptomic analyses reveal minor-yet-crucial roles of gut microbiome in deep-sea hydrothermal vent snail
Source: Anim Microbiome. 2022 Jan 3;4:3. doi: 10.1186/s42523-021-00150-z (PMC8722025; doi:10.1186/s42523-021-00150-z)
Supplement: Supplementary file 1 — Additional file 1. Figure S1. Rarefaction curves for gut microbial samples of three Alviniconcha marisindica individuals from the Wocan vent field. Figure S2. Gene Ontology (GO) enrichment network of differentially expressed genes (DEGs). The significantly (p-value < 0.01) enriched GO terms of selected highly expressed genes in the intestine of Alviniconcha marisindica are clustered in accordance with their functional category. The connecting pairs of nodes showing the intra-cluster and inter-cluster similarities of enriched terms. The colour code represents different cluster annotations. Each node represents an enriched term. Figure S3. Differentially expressed genes (DEGs) in the intestine and gill of Alviniconcha marisindica. Volcano plot and biological coefficient of variation (BCV) plot of DEGs in the intestine are identified by DESeq2 analysis. The log10 (FDR corrected p-values) are plotted against the log2 (FC) in gene expression. Upregulated genes by twofold or more and with a FDR corrected p-value < 0.05 are marked as blue dots, whilst down-regulated genes (FRD ≤ 2 with P < 0.05) are marked in red colour. Figure S4. Photograph of snail Alviniconcha marisindica collected from the Wocan hydrothermal field (WHF) and stored in absolute ethanol. Figure S5. SEM images of radula. Overview: (a) Alviniconcha marisindica (individual 01); scale bar = 300 μm (b) A. marisindica (individual 01); scale bars = 200 μm. Central and lateral teeth close-up: (c) A. marisindica (individual 01); scale bars = 200 μm (d) A. marisindica (individual 02); scale bars = 200 μm. Marginal teeth close-up: (e) A. marisindica (individual 02); scale bars = 30 μm. [file 42523_2021_150_MOESM1_ESM.pdf]

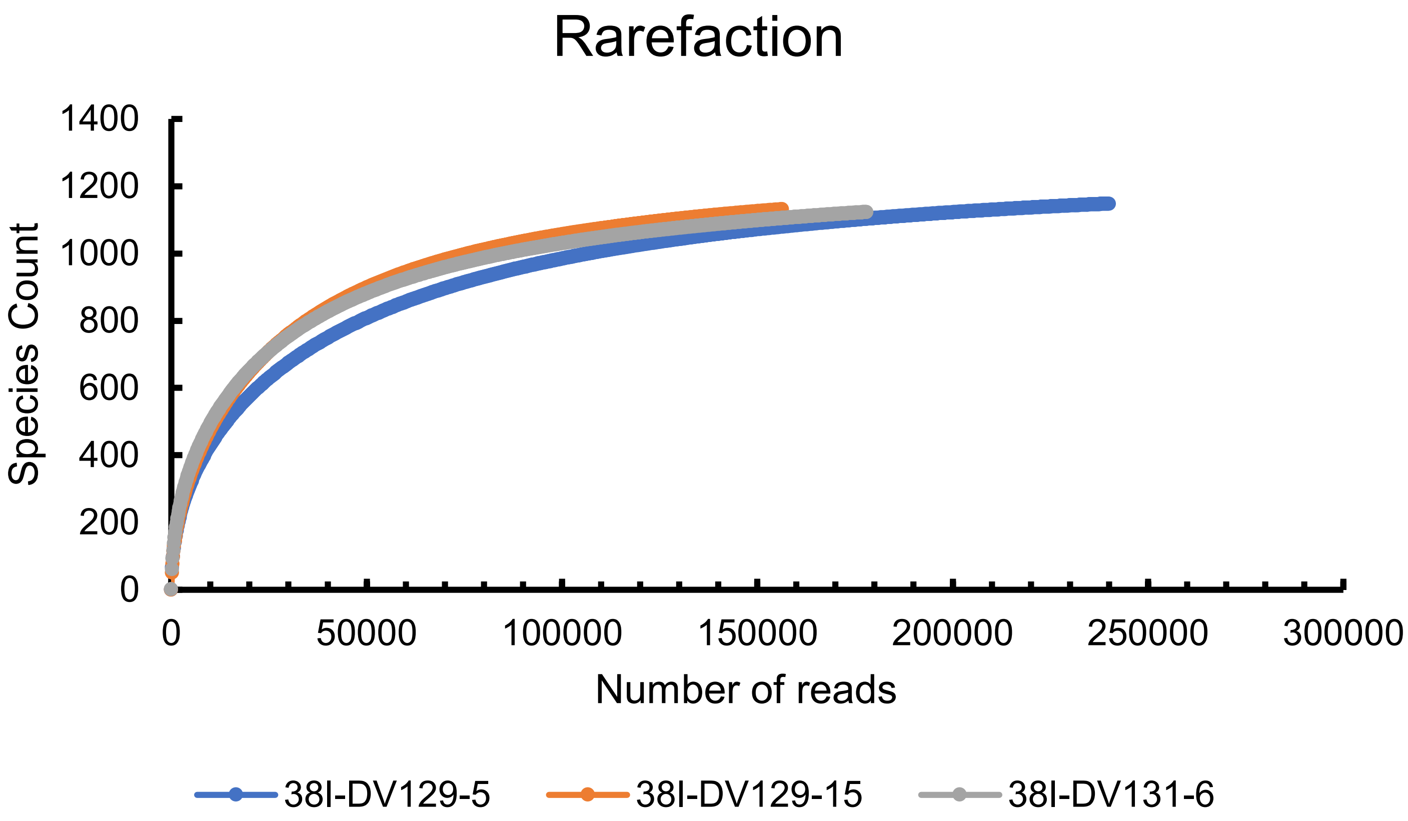
Rarefaction
1400
1200
1000
800
600
400
200
0
Species Count
0
50000
100000
150000
200000
250000
300000
Number of reads
38I-DV129-5
38I-DV129-15
38I-DV131-6

Figure S1

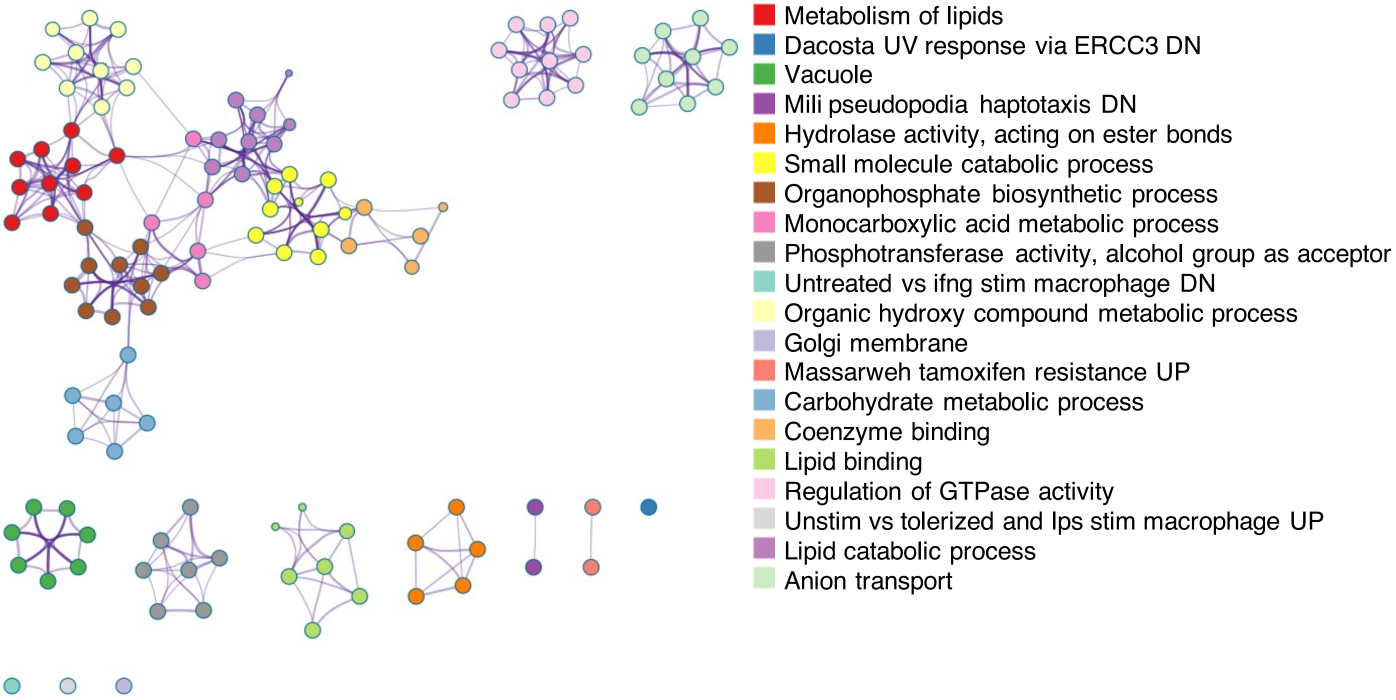

Metabolism of lipids
Dacosta UV response via ERCC3 DN
Vacuole
Mili pseudopodia haptotaxis DN
Hydrolase activity, acting on ester bonds
Small molecule catabolic process
Organophosphate biosynthetic process
Monocarboxylic acid metabolic process
Phosphotransferase activity, alcohol group as acceptor
Untreated vs ifng stim macrophage DN
Organic hydroxy compound metabolic process
Golgi membrane
Massarweh tamoxifen resistance UP
Carbohydrate metabolic process
Coenzyme binding
Lipid binding
Regulation of GTPase activity
Unstim vs tolerized and lps stim macrophage UP
Lipid catabolic process
Anion transport

Figure S2

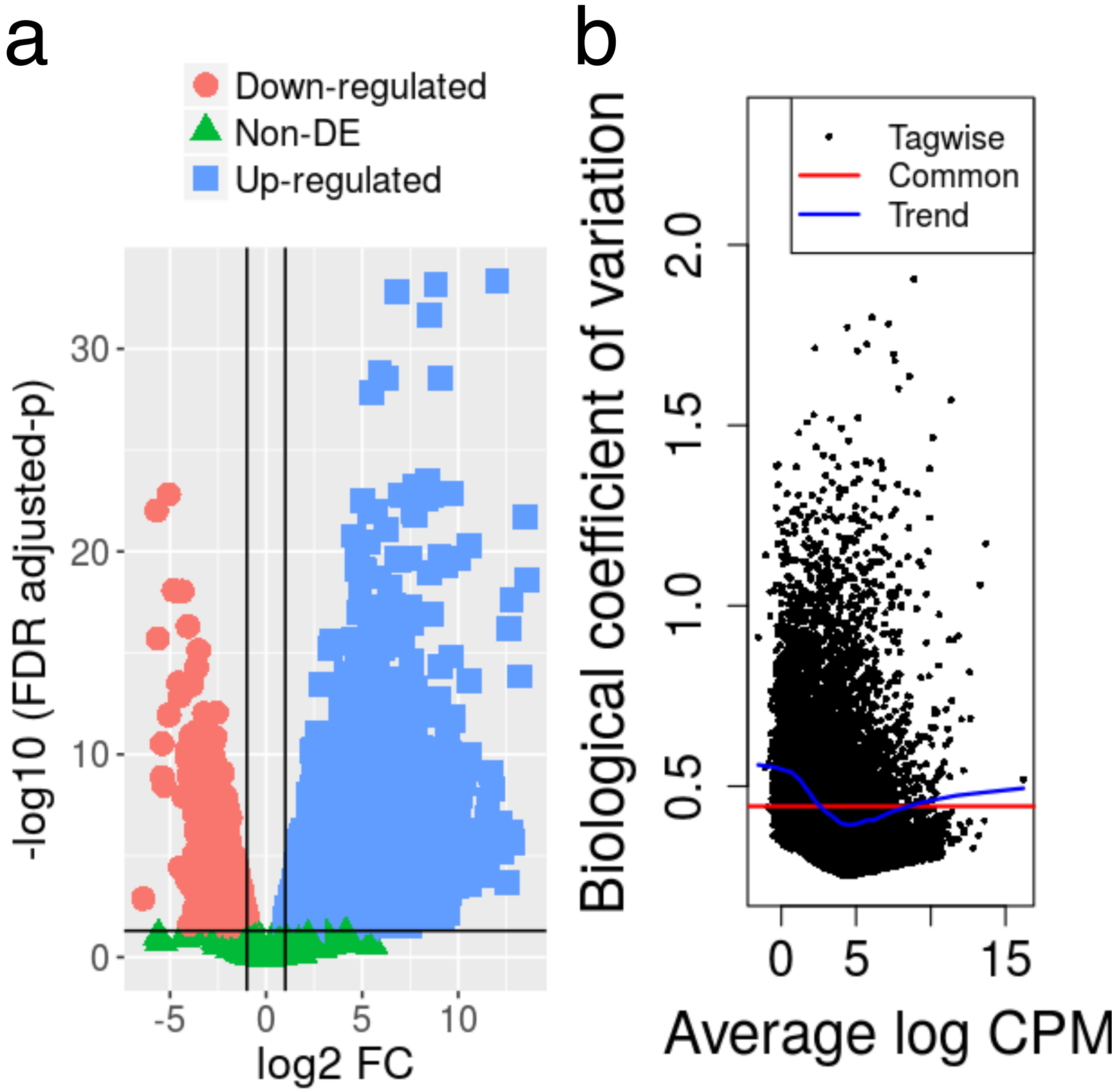
a
Down-regulated
Non-DE
Up-regulated
-log10 (FDR adjusted-p)
30
20
10
0
-5
0
5
10
log2 FC
b
Biological coefficient of variation
Tagwise
Common
Trend
2.0
1.5
1.0
0.5
0
5
15
Average log CPM

Figure S3

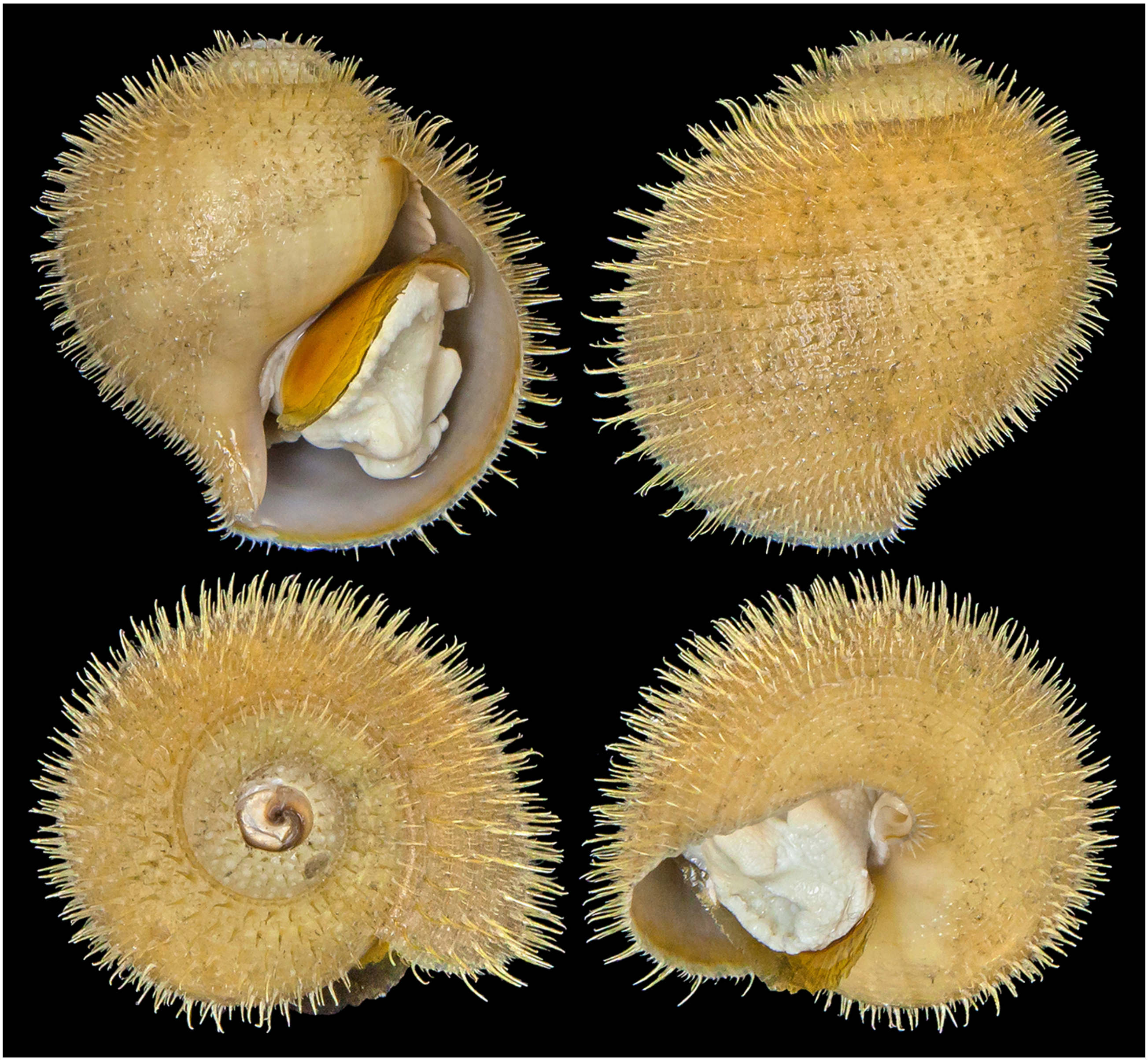

Figure S4

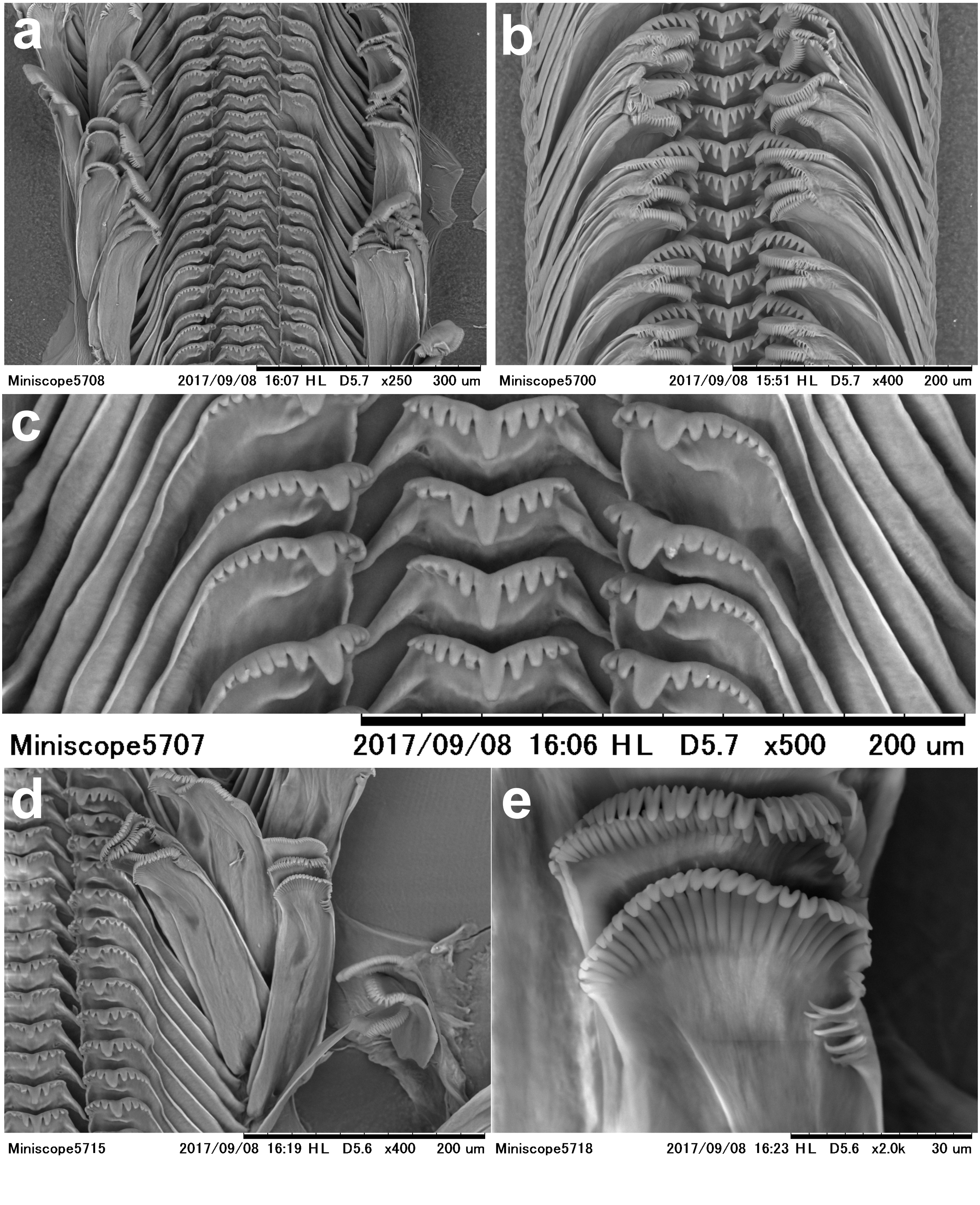
a
b
c
d
e
Miniscope5708
2017/09/08 16:07 HL D5.7 x250 300 um
Miniscope5700
2017/09/08 15:51 HL D5.7 x400 200 um
Miniscope5707
2017/09/08 16:06 HL D5.7 x500 200 um
Miniscope5715
2017/09/08 16:19 HL D5.6 x400 200 um
Miniscope5718
2017/09/08 16:23 HL D5.6 x2.0k 30 um

Figure S5
