# Supplementary material for: Metagenomic and metatranscriptomic analyses reveal minor-yet-crucial roles of gut microbiome in deep-sea hydrothermal vent snail
Source: Anim Microbiome. 2022 Jan 3;4:3. doi: 10.1186/s42523-021-00150-z (PMC8722025; doi:10.1186/s42523-021-00150-z)
Supplement: Supplementary file 2 — Additional file 2. Table S1. Metagenomic and 16S rRNA amplicon data of intestines of Alviniconcha marisindica from the Wocan field, intestines of giant land snails, intestines of deep-sea bone-eating, scavenger and predatory snails and environmental samples from deep-sea habitats. Table S2. Annotation and expression levels of genes involved in encoding representative exo-hydrolases in intestinal microbiomes of three A. marisindica individuals from the Wocan site. [file 42523_2021_150_MOESM2_ESM.docx]

**Table S1.** Metagenomic and 16S rRNA amplicon data of intestines of *Alviniconcha marisindica* from the Wocan field, intestines of giant land snails, intestines of deep-sea bone-eating, scavenger and predatory snails and environmental samples from deep-sea habitats.

| **ID** | **Sequence method** | **Sequence type** | **Animals/Environment** | **Material** | **Location** | **Depth (m)** |
| --- | --- | --- | --- | --- | --- | --- |
| **SRR11781645** | Illumina | WGS | *Alviniconcha marisindica* | Intestine | Carlsberg Ridge | 2,919 |
| **SRR11781642** | Illumina | WGS | *Alviniconcha marisindica* | Intestine | Carlsberg Ridge | 2,919 |
| **SRR11781639** | Illumina | WGS | *Alviniconcha marisindica* | Intestine | Carlsberg Ridge | 2,919 |
| **mgm4482672.3** | 454 | WGS | *Achatina fulica* | Intestine | Rio de Janeiro | _ |
| **SRR9166292** | Illumina | Amplicon 16S V3V4 | *Pomacea canaliculata* | Intestine | Nanheng River, Shanghai | _ |
| **SRR9166293** | Illumina | Amplicon 16S V3V4 | *Pomacea canaliculata* | Intestine | Nanheng River, Shanghai | _ |
| **SRR9166294** | Illumina | Amplicon 16S V3V4 | *Pomacea canaliculata* | Intestine | Nanheng River, Shanghai | _ |
| **dryad.5h1q1** | Illumina | Amplicon 16S V4 | *Bathymargarites symplector* | Intestine | Gulf of California | 3,651 |
| **dryad.5h1q1** | Illumina | Amplicon 16S V4 | *Phymorhynchus* sp. | Intestine | Gulf of California | 3,651 |
| **dryad.5h1q1** | Illumina | Amplicon 16S V4 | *Rubyspira osteovora* | Intestine | Monterey Submarine Canyon | 2,893 |
| **dryad.5h1q1** | Illumina | Amplicon 16S V4 | *Rubyspira osteovora* | Intestine | Monterey Submarine Canyon | 2,893 |
| **dryad.5h1q1** | Illumina | Amplicon 16S V4 | *Rubyspira osteovora* | Intestine | Monterey Submarine Canyon | 2,893 |
| **dryad.5h1q1** | Illumina | Amplicon 16S V4 | *Rubyspira osteovora* | Intestine | Monterey Submarine Canyon | 2,893 |
| **dryad.5h1q1** | Illumina | Amplicon 16S V4 | Environment | Sediment | Monterey Submarine Canyon | 2,893 |
| **dryad.5h1q1** | Illumina | Amplicon 16S V4 | Environment | Whale bone | Monterey Submarine Canyon | 2,893 |
| **mgm4448226.3** | 454 | WGS | Environment | Water | Mariana Trough | 2,850 |
| **mgm4773053.3** | Illumina | MT | Environment | Sediment microbial mat | East Pacific Rise | 2,506 |
| **mgm4773054.3** | Illumina | WGS | Environment | Water | East Pacific Rise | 2,506 |

**Table S2.** Annotation and expression levels of genes involved in encoding representative exo-hydrolases in intestinal microbiomes of three *A. marisindica* individuals from the Wocan site.

| **Individual 38I-DV129-5** | | |  |  |  |
| --- | --- | --- | --- | --- | --- |
| **Gene_ID** | **Annotation** | **Gene** | **COG** | **Best Tax-Level** | **TPM** |
| **k141_240474_2** | sialate O-acetylesterase-like | | G | Cytophagia | 283.14 |
| **k141_338559_2** | sialate O-acetylesterase | | G | Leeuwenhoekiella | 122.07 |
| **k141_402846_1** | sialate O-acetylesterase-like | | G | Sphingobacteriia | 61.69 |
| **k141_216833_3** | Alpha amylase, catalytic domain | treY | G | Microbacteriaceae | 490.45 |
| **k141_34583_1** | Alpha-amylase domain | | G | Sphingomonadales | 304.57 |
| **k141_698499_2** | maltase A2-like | | G | Lactobacillaceae | 328.87 |
| **k141_698499_2** | maltase A2-like | | G | Lactobacillaceae | 702.71 |
| **k141_240474_2** | sialate O-acetylesterase-like | | G | Cytophagia | 262.29 |
| **k141_338559_2** | sialate O-acetylesterase | | G | Leeuwenhoekiella | 112.56 |
| **k141_402846_1** | sialate O-acetylesterase-like | | G | Sphingobacteriia | 4050.85 |
| **k141_240474_2** | sialate O-acetylesterase-like | | G | Cytophagia | 262.29 |
| **k141_216833_3** | Alpha amylase, catalytic domain | treY | G | Microbacteriaceae | 30.38 |
| **k141_295070_4** | alpha-glucosidase | aglA | G | Myxococcales | 46.35 |
| **k141_34583_1** | Alpha-amylase domain | | G | Sphingomonadales | 232.23 |
| **k141_188836_2** | GDSL-like Lipase/Acylhydrolase family | | E | Cytophagia | 18.91 |
| **k141_283355_1** | GDSL-like Lipase/Acylhydrolase family | | E | Cytophagia | 1.65 |
| **k141_513053_1** | GDSL-like Lipase/Acylhydrolase family | tesA | E | Leeuwenhoekiella | 36.49 |
| **k141_60153_1** | GDSL-like Lipase/Acylhydrolase | | E | Micromonosporales | 45.59 |
| **k141_479102_1** | PFAM GDSL-like Lipase Acylhydrolase | | E | Nostocales | 1.55 |
| **k141_299134_1** | GDSL-like Lipase/Acylhydrolase family | | E | Proteobacteria | 10.65 |
| **k141_747374_1** | G-D-S-L family lipolytic protein | | E | Proteobacteria | 6.50 |
| **k141_435642_1** | GDSL-like Lipase/Acylhydrolase family | | E | Sphingomonadales | 156.20 |
| **k141_572192_2** | GDSL-like Lipase/Acylhydrolase family | | E | Sphingomonadales | 43.11 |
| **k141_72843_1** | GDSL-like Lipase/Acylhydrolase family | | E | Streptosporangiales | 4.95 |
| **k141_813635_1** | Belongs to the glycosyl hydrolase 8 (cellulase D) family | | G | Cytophagia | 2.54 |
| **k141_371312_1** | Cellulase (glycosyl hydrolase family 5) | | G | Cellvibrio | 16.81 |
| **k141_717242_2** | Cellulase EGX3 | | G | Paenibacillaceae | 3.95 |
| **k141_499802_1** | carboxylesterase 3B | lipT | I | Gordoniaceae | 383.44 |
| **k141_344000_3** | para-nitrobenzyl esterase | pnbA | I | Paenibacillaceae | 1.55 |
| **k141_219741_3** | esterase E4-like | | I | Sphingomonadales | 114.20 |
| **k141_175913_1** | Exodeoxyribonuclease III | | L | Bacteria | 98.89 |
| **k141_113311_7** | Exodeoxyribonuclease III | | L | Bacteria | 47.97 |
| **k141_8172_1** | Exodeoxyribonuclease III | | L | Bacteria | 4.49 |
| **k141_40481_1** | Exodeoxyribonuclease III | | S | Proteobacteria | 7.73 |
| **k141_262990_1** | Exodeoxyribonuclease III | holA | L | Tenericutes | 608.82 |
| **k141_175913_1** | Exodeoxyribonuclease III | | L | Bacteria | 98.89 |
| **k141_39157_1** | Trypsin |  | E | Corynebacteriaceae | 2420.31 |
| **k141_791929_1** | Trypsin-like serine protease | | M | Vibrionales | 33.11 |
| **k141_39157_1** | Trypsin |  | E | Corynebacteriaceae | 2420.31 |
| **k141_562146_2** | neutral protease-like | lasB | Q | Colwelliaceae | 2.96 |
| **k141_541590_1** | neutral protease-like | lasB | E | Vibrionales | 75.77 |
| **k141_562146_2** | neutral protease-like | lasB | Q | Colwelliaceae | 2.96 |
| **k141_46506_1** | Papain-like cysteine protease AvrRpt2 | | H | Bacillus | 1557.63 |
| **k141_46506_1** | Papain-like cysteine protease AvrRpt2 | | H | Bacillus | 1557.63 |
| **k141_824392_1** | hemagglutinin | lasB | E | Shewanellaceae | 1.56 |
| **k141_776239_3** | Protease prsW family | | S | Dermatophilaceae | 305.60 |
| **Individual 38I-DV129-15** | | |  |  |  |
| **k141_688074_2** | sialate O-acetylesterase-like | | G | Cytophagia | 1.91 |
| **k141_543608_2** | sialate O-acetylesterase | | G | Leeuwenhoekiella | 47.14 |
| **k141_42238_1** | SMART alpha amylase catalytic sub domain | | G | Chloroflexi | 324.57 |
| **k141_352388_1** | SMART alpha amylase catalytic sub domain | | G | Chloroflexi | 115.94 |
| **k141_819448_3** | Maltogenic Amylase, C-terminal domain | treS | G | Rhizobiaceae | 534.59 |
| **k141_236157_3** | 1,4-alpha-glucan-branching enzyme | GLC3 | G | Chaetomiaceae | 178.97 |
| **k141_908504_2** | GDSL-like Lipase/Acylhydrolase | | E | Alteromonadaceae | 16.04 |
| **k141_660256_1** | GDSL-like Lipase/Acylhydrolase family | ypmR | E | Carnobacteriaceae | 51.37 |
| **k141_318019_1** | GDSL-like Lipase/Acylhydrolase family | | E | Cytophagia | 0.76 |
| **k141_672619_1** | GDSL-like Lipase/Acylhydrolase family | | E | Flavobacteriia | 6.04 |
| **k141_366024_1** | GDSL-like Lipase/Acylhydrolase family | tesA | E | Leeuwenhoekiella | 0.97 |
| **k141_637991_1** | GDSL-like Lipase/Acylhydrolase | | E | Micromonosporales | 372.32 |
| **k141_519721_1** | GDSL-like Lipase/Acylhydrolase | | E | Micromonosporales | 66.99 |
| **k141_781927_1** | GDSL-like Lipase/Acylhydrolase | | E | Micromonosporales | 59.82 |
| **k141_639040_1** | Belongs to the glycosyl hydrolase 8 (cellulase D) family | | G | Cytophagia | 4.33 |
| **k141_540585_8** | Cellulase (glycosyl hydrolase family 5) | | G | Cellvibrio | 6.74 |
| **k141_39007_1** | PFAM GDSL-like Lipase Acylhydrolase | | E | Nostocales | 8.19 |
| **k141_640620_1** | PFAM GDSL-like Lipase Acylhydrolase | | E | Nostocales | 2.14 |
| **k141_353962_1** | GDSL-like Lipase/Acylhydrolase family | | S | Pleosporales | 15.63 |
| **k141_302376_2** | GDSL-like Lipase/Acylhydrolase | | E | Porphyromonadaceae | 3.02 |
| **k141_319940_1** | GDSL-like Lipase/Acylhydrolase family | | E | Sphingomonadales | 39.71 |
| **k141_368234_1** | GDSL-like Lipase/Acylhydrolase family | | E | Streptosporangiales | 1.30 |
| **k141_908504_2** | GDSL-like Lipase/Acylhydrolase | | E | Alteromonadaceae | 16.04 |
| **k141_463823_1** | Esterase FE4 | | G | Ascomycota | 39.42 |
| **k141_542556_1** | Exodeoxyribonuclease III | | G | Ascomycota | 2.70 |
| **k141_664301_1** | Belongs to the type-B carboxylesterase lipase family | | I | Eurotiales | 48.53 |
| **k141_238923_1** | carboxylesterase 3B | lipT | I | Gordoniaceae | 83.59 |
| **k141_712297_1** | Exodeoxyribonuclease III | | | Agaricomycetes incertae sedis | 0.75 |
| **k141_542556_1** | Exodeoxyribonuclease III | | G | Ascomycota | 2.70 |
| **k141_621930_1** | Exodeoxyribonuclease III | | L | Bacteria | 110.90 |
| **k141_611192_1** | Exodeoxyribonuclease III | | L | Bacteria | 13.32 |
| **k141_49768_1** | Exodeoxyribonuclease III | | L | Bacteria | 6.64 |
| **k141_489964_2** | Exodeoxyribonuclease III | | L | Bacteria | 4.03 |
| **k141_322147_1** | Exodeoxyribonuclease III | ypmS | S | Listeriaceae | 19.94 |
| **k141_448732_3** | Exodeoxyribonuclease III | exoA | L | Neisseriales | 3.38 |
| **k141_560323_2** | Exodeoxyribonuclease III | | S | Proteobacteria | 405.28 |
| **k141_711647_1** | Exodeoxyribonuclease III | NAR1 | Y | Taphrinomycotina | 22.07 |
| **k141_396224_1** | fibrinolytic enzyme, isozyme C-like | | O | Actinobacteria | 139.91 |
| **k141_246661_1** | chymotrypsin-like serine proteinase | | O | Actinobacteria | 3.24 |
| **k141_2041_1** | Trypsin |  | E | Corynebacteriaceae | 613.73 |
| **k141_396224_1** | fibrinolytic enzyme, isozyme C-like | | O | Actinobacteria | 139.91 |
| **k141_246661_1** | chymotrypsin-like serine proteinase | | O | Actinobacteria | 3.24 |
| **k141_686364_2** | neutral protease-like | lasB | Q | Colwelliaceae | 0.40 |
| **k141_462614_1** | neutral protease-like | lasB | E | Gammaproteobacteria | 8.32 |
| **k141_148422_1** | neutral protease-like | lasB | E | Vibrionales | 15.26 |
| **k141_462614_1** | neutral protease-like | lasB | E | Gammaproteobacteria | 8.32 |
| **k141_825267_1** | Papain-like cysteine protease AvrRpt2 | | S | Clostridia | 4.42 |
| **k141_605305_1** | Papain-like cysteine protease AvrRpt2 | | S | Clostridia | 2.49 |
| **k141_534720_1** | Papain-like cysteine protease AvrRpt2 | | S | Clostridia | 1.73 |
| **k141_470447_1** | Papain-like cysteine protease AvrRpt2 | | S | Clostridia | 1.10 |
| **k141_726180_1** | Papain-like cysteine protease AvrRpt2 | | S | Clostridia | 1.06 |
| **k141_686583_1** | Papain-like cysteine protease AvrRpt2 | | S | Clostridia | 0.87 |
| **k141_417641_4** | Protease prsW family | | S | Dermatophilaceae | 64.06 |
| **k141_890620_1** | matrilysin family metalloendoprotease | | O | Proteobacteria | 35.72 |
| **k141_847292_1** | OTU-like cysteine protease | | OT | Eurotiales | 5.79 |
| **Individual 38I-DV131-6** | | |  |  |  |
| **k141_623736_1** | sialate O-acetylesterase | | G | Leeuwenhoekiella | 27.19 |
| **k141_469403_1** | sialate O-acetylesterase-like | | G | Sphingobacteriia | 2004.59 |
| **k141_366488_2** | SMART alpha amylase catalytic sub domain | | G | Chloroflexi | 85.31 |
| **k141_758050_2** | maltase A2-like | | G | Lactobacillaceae | 226.31 |
| **k141_513093_1** | maltase A3-like | | G | Leuconostocaceae | 1.54 |
| **k141_469403_1** | sialate O-acetylesterase-like | | G | Sphingobacteriia | 2004.59 |
| **k141_461237_1** | alpha-glucosidase | malZ | G | Oceanospirillales | 14.50 |
| **k141_474653_5** | 1,4-alpha-glucan-branching enzyme | GLC3 | G | Chaetomiaceae | 466.84 |
| **k141_366488_2** | SMART alpha amylase catalytic sub domain | | G | Chloroflexi | 85.31 |
| **k141_511492_1** | GDSL-like Lipase/Acylhydrolase | | E | Alteromonadaceae | 12.12 |
| **k141_290837_1** | GDSL-like Lipase/Acylhydrolase | | E | Clostridia | 2.77 |
| **k141_454472_2** | Lysophospholipase L1 | | E | Cytophagia | 11.80 |
| **k141_652332_1** | hydrolase GDSL | | E | Cytophagia | 3.40 |
| **k141_566963_1** | GDSL-like Lipase/Acylhydrolase family | | E | Flavobacteriia | 41.46 |
| **k141_23211_1** | GDSL-like Lipase/Acylhydrolase family | | E | Hyphomonadaceae | 484.00 |
| **k141_483011_1** | GDSL-like Lipase/Acylhydrolase | | E | Micromonosporales | 152.28 |
| **k141_445749_1** | GDSL-like Lipase/Acylhydrolase | | E | Micromonosporales | 40.19 |
| **k141_599789_1** | GDSL-like Lipase/Acylhydrolase | estA | E | Sphingobacteriia | 12.80 |
| **k141_553159_2** | GDSL-like Lipase/Acylhydrolase family | | E | Sphingomonadales | 37.03 |
| **k141_546352_1** | G-D-S-L family lipolytic protein | | E | Sphingomonadales | 4.12 |
| **k141_511492_1** | GDSL-like Lipase/Acylhydrolase | | E | Alteromonadaceae | 12.12 |
| **k141_273217_1** | Belongs to the glycosyl hydrolase 8 (cellulase D) family | | G | Cytophagia | 6.08 |
| **k141_545084_1** | Cellulase (glycosyl hydrolase family 5) | | G | Cellvibrio | 7.56 |
| **k141_416738_1** | Cellulase EGX3 | | G | Paenibacillaceae | 448.70 |
| **k141_173438_1** | Belongs to the glycosyl hydrolase 8 (cellulase D) family | | I | Gammaproteobacteria | 769.77 |
| **k141_456679_1** | Belongs to the glycosyl hydrolase 8 (cellulase D) family | | I | Bacteroidetes | 357.21 |
| **k141_378516_2** | Belongs to the type-B carboxylesterase lipase family | | I | Actinobacteria | 32.96 |
| **k141_776825_1** | para-nitrobenzyl esterase | pnbA | I | Bacillus | 45.32 |
| **k141_564754_1** | Belongs to the type-B carboxylesterase lipase family | | I | Eurotiales | 107.07 |
| **k141_568994_1** | carboxylesterase 3B | lipT | I | Gordoniaceae | 360.87 |
| **k141_785481_1** | Belongs to the type-B carboxylesterase lipase family | lipT | I | Mycobacteriaceae | 209.83 |
| **k141_166509_1** | Belongs to the type-B carboxylesterase lipase family | | T | Nectriaceae | 26.71 |
| **k141_378516_2** | Belongs to the type-B carboxylesterase lipase family | | I | Actinobacteria | 32.96 |
| **k141_62324_7** | Exodeoxyribonuclease III | exoA | L | Alteromonadaceae | 12.69 |
| **k141_603633_1** | Exodeoxyribonuclease III | | L | Bacteria | 9.79 |
| **k141_354178_2** | Exodeoxyribonuclease III | | L | Bacteria | 6.34 |
| **k141_199648_2** | Exodeoxyribonuclease III | | L | Bacteria | 5.53 |
| **k141_25149_1** | Exodeoxyribonuclease III | ypmS | S | Listeriaceae | 80.29 |
| **k141_46566_1** | Exodeoxyribonuclease III | | S | Proteobacteria | 3.20 |
| **k141_283492_1** | Exodeoxyribonuclease III | NAR1 | Y | Taphrinomycotina | 32.33 |
| **k141_281926_1** | Exodeoxyribonuclease III | holA | L | Tenericutes | 108.10 |
| **k141_62324_7** | Exodeoxyribonuclease III | exoA | L | Alteromonadaceae | 12.69 |
| **k141_31145_1** | Trypsin |  | E | Corynebacteriaceae | 303.71 |
| **k141_111587_4** | neutral protease-like | lasB | Q | Colwelliaceae | 2.54 |
| **k141_385692_1** | neutral protease-like | lasB | E | Gammaproteobacteria | 1.76 |
| **k141_588201_1** | neutral protease-like | lasB | E | Vibrionales | 10.02 |
| **k141_111587_4** | neutral protease-like | lasB | Q | Colwelliaceae | 2.54 |
| **k141_46789_4** | Papain-like cysteine protease AvrRpt2 | | H | Bacillus | 8.81 |
| **k141_445402_1** | peptidase domain protein | | S | Clostridia | 1.71 |
| **k141_660310_1** | Papain-like cysteine protease AvrRpt2 | | S | Clostridia | 0.74 |
| **k141_534044_1** | Papain-like cysteine protease AvrRpt2 | | S | Clostridia | 0.68 |
| **k141_488656_1** | Papain-like cysteine protease AvrRpt2 | | S | Clostridia | 0.65 |
| **k141_774423_1** | Papain-like cysteine protease AvrRpt2 | | S | Clostridia | 0.44 |
| **k141_46789_4** | Papain-like cysteine protease AvrRpt2 | | H | Bacillus | 8.81 |
| **k141_759841_8** | Protease prsW family | | S | Dermatophilaceae | 113.86 |
